# Supplementary material for: Barriers and facilitators to addressing mental health needs among Asian, Black and Latin American men who have sex with men (MSM) in England and Wales: A qualitative study
Source: PLOS Ment Health. 2025 Mar 27;2(3):e0000231. doi: 10.1371/journal.pmen.0000231 (PMC12798512; doi:10.1371/journal.pmen.0000231)
Supplement: S1 File — Interview topic guide. (DOCX) [file pmen.0000231.s001.docx]

**S1 File: Interview topic guide**

1. **Introductions**

- Researcher, Sigma, LSHTM
- This is an interview to hear your thoughts on HIVST. You’ve been invited because of your involvement with the SELPHI study. I’ll ask you some specific questions about yourself, and some questions about your history. Feel free to answer them however you see fit. There are no right or wrong answers etc.

1. **Ethics**

- We would like to record the interview so we don’t miss any of what you say.
- Explain how we will use and protect the data
- Do you have any questions?
- Are you happy to proceed?

1. **HIV testing history**

- Had you tested for HIV before you joined SELPHI?

*If no*

- Have you considered HIV testing before?
- What has stopped you?
- Have you ever considered testing but not gone?
- What do your friends think about you not testing for HIV?
- Can you describe what norms there are generally in the gay community around testing?
- The last time you had a risk, did you consider going for an HIV test? Describe your decision making process around that? Why did you NOT test?
- What risk would you have to take to go for a test?
- Have you ever tested for STIs?

***Once finished go to section 5***

*If yes*

- Can you tell me about the first time you tested for HIV?
- Can you remember what prompted you to seek testing the first time you went?
- What was the experience like? Where did you go? How did you choose to test that way?
- What kind of support were you provided with? Was this what you wanted?
- Can you think of anything else that would have been helpful for you in that situation?
- How about the last time you tested before SELPHI? Can you tell me a bit more about that?
- Where did you go? How did you make that decision? What kind of support did you get?

1. **HIV testing patterns (If previously tested)**

- What is usually your motivation to test? Do you have conversations about HIV testing with your friends? Do your friends support each other around HIV testing?
- How often do you test for HIV typically? Do you always get an STI screen when you test? (*prompt:* Do you have a testing routine?)
- What challenges have you faced when taking a test or thinking about testing? Are there things that have stopped you from testing? Or that make testing more difficult?
- What are your feelings about having a healthcare worker of a similar ethnic background to yourself? How about of a similar sexual orientation?
- What, if any, experiences of racism or homophobia have you had when visiting a clinic? How about other negative experiences in clinics?
- What about in healthcare settings more generally? Can you describe what happened?
- The last time you had a risk, did you consider going for an HIV test? Describe your decision making process around that? Where did you go? What role does risk play for you generally in testing for HIV?

(*if not answered*) what kind of risk would prompt you to look for an HIV test?

- Has there ever been a time you tested when you were genuinely concerned you may be HIV positive? What made you think that? How did you test and what led you to choose that method? Is that the same thing you would do now?

1. **Mental health**

- What types of conversations do you have with your friends and family about mental health? What about with other gay / bi guys? Can you tell me how these similar or different?
- Have you ever had an issues with your mental health? Can you tell me about what happened?

*If yes*

- What kind of support did you access?
- How did you choose that service? Did it meet the needs you had at the time?
- Would you choose to go to a similar or different service in the future? Why?
- Has your mental health ever impacted on drinking, taking drugs or your sexual behaviour? If so, in what ways?

*If no*

- If you did feel like you wanted support around your mental health, where would you go? How would you make decisions about the type of service you would want to access?

1. **Engagement with HIVST & SELPHI**

- When did you first hear about HIVST?
- What were your initial thoughts?
- How did you hear about the SELPHI study? What make you decide to take part?
- Do you remember the advert for the study that you saw? Anything stick in your mind from that?
- **[Show study advert]** Here’s the advert you were recruited from. What do you think about it? Is there anything you find appealing or not about it? Was it motivational in any way?
- How did you find the process of signing up? Was there anything difficult? Anything that you didn’t understand?
- **[Show registration and enrolment surveys (*Filename: surveys 1*)]** Here are the surveys that you filled out at the time. What jumps out in your mind about these?
- Were there any of these questions unclear when you completed the surveys? Any that you didn’t feel comfortable answering?
- How long did it take for your kit to arrive?

1. **Experience of HIVST & trial infrastructure**

- **[Show kit with accompanying sleeve]** This is the same version of the test we sent you. Can you remember what your first impressions of it were? (Make sure sleeve and kit itself are covered in conversation)
- Thinking back to when you first took the self-test, what was going through your mind? Tell me everything you can about the first time you used HIVST (*Prompt*: Where were you? did you think there was a possibility of a positive result?).
- Did you decide to take the test by yourself or was there someone with you?
- Did you read the instructions? How many times?
  (*Prompt:* if not answered: What did you make of them? Did you watch any of the videos online? Did the kit look easy to use?)
- How was your overall experience of using the test kit?
  (*Prompt*: Do you think you made any mistakes? Was it difficult to use the lancet?)
- Describe your experience of reading the result.
- How did you feel after using HIVST? Did you trust the result that your test gave you?
- Did you seek support from anywhere?
- Have you talked to anyone about HIVST? Did you tell anyone you had taken a self-test? What did you tell them about the experience? What do you think they thought about it?
- Do you remember receiving a follow-up survey about two weeks after you took the test? What were your impressions?
- **[Show copy of email (*filename: email 1*) and two week surveys (*Filename: survey 2*)]** This is what it would have looked like. Can you think about how you reacted to this? (prompt: did you recognise this as a form of support?)
- **[Show copy of email (*filename: email 2*) and three month survey (*Filename: survey 3*)]** Do you remember receiving this survey? What was your initial reaction to the email? Did you fill the survey out?

1. **Experiences of randomisation B *(if randomised to RT)***

- So you were randomised to receive repeat testing at your three month point.
- How have you felt about the offer of repeat testing? Has it been useful? How many tests did you have through SELPHI? Did you use all the tests that have been offered? (if not used) what has influenced you to not take a test? Did you share tests?
- Did you also access other testing options?
- **[Show copy of email (*filename: email 3*) and three month survey (*Filename: survey 4*)]** Were these emails been useful in prompting you for more testing?
- Were there changes to the way you think about testing through this process?

1. **After HIVST**

- What are your thoughts on using an oral fluid test rather than blood?
- On reflection, is there any additional support you would want with an HIVST?
- Are there any changes we could make to the self-testing offer that would make it easier for people to use self-testing?
- Do you think your behaviour will change in any way following the experience of using HIVST?
- Would you use HIVST again if it was available to you? Would you use it alongside other services or instead?
- Is there someone or a group of people you think HIVST is particularly good for? Anyone that it is not suitable for?

1. **Experiences of the scene / racism *(ask if race / ethnicity is under discussed in preceding sections)***

- What would you say the size of your friendship group is?
- Can you tell me about your friendship group? Are they pretty mixed in terms of age / ethnicity?
- Do you spend much time on the ‘scene’? What have your experiences with the ‘scene’ been like?
- Have you faced any racism when accessing the scene, either online or in person?
- How, if at all, do these experiences shape the way you thinking about HIV testing or mental health?
- Do you feel like HIV testing / mental health services (*delete as appropriate*) are designed with you in mind? How so?
- Is there anything else you’d like to discuss?
