# Supplementary material for: Barriers and facilitators to addressing mental health needs among Asian, Black and Latin American men who have sex with men (MSM) in England and Wales: A qualitative study
Source: PLOS Ment Health. 2025 Mar 27;2(3):e0000231. doi: 10.1371/journal.pmen.0000231 (PMC12798512; doi:10.1371/journal.pmen.0000231)
Supplement: Attachment — Submitted filename: Ghobrial_responses_R2.docx [file pmen.0000231.s003.docx]

**Responses to reviewer comments**

Dear Prof Reyes,

Thank you for the further reviewers’ comments for this article. We have addressed the new comments point by point and include a new tracked version of the manuscript.

Thank you,

Dr Andrew Ghobrial

_________________________________________________________

Journal Requirements:

1. Please review your reference list to ensure that it is complete and correct. If you have cited papers that have been retracted, please include the rationale for doing so in the manuscript text, or remove these references and replace them with relevant current references. Any changes to the reference list should be mentioned in the rebuttal letter that accompanies your revised manuscript. If you need to cite a retracted article, indicate the article’s retracted status in the References list and also include a citation and full reference for the retraction notice.

*We have reviewed the reference list and ensured that it is compliant with APA guidelines by making minor formatting changes.*

*We have also added the following references to support our response to reviewers comments:*

*Roy, M. (2024). A trail leading home: Analysing the evolution of Mpox risk narratives and targets of blame in UK media. Health, Risk & Society, 26(3–4), 115–134.*

*Witzel, T. C., Ghobrial, A., Palich, R., Charles, H., Rodger, A. J., Sabin, C., Sparrowhawk, A., Pool, E. R., Prochazka, M., & Vivancos, R. (2024). Experiences of mpox illness and case management among cis and trans gay, bisexual, and other men who have sex with men in England: A qualitative study. EClinicalMedicine, 70, 101553. https://doi.org/10.1016/j.eclinm.2024.101553*

6. Review Comments to the Author

Please use the space provided to explain your answers to the questions above. You may also include additional comments for the author, including concerns about dual publication, research ethics, or publication ethics. (Please upload your review as an attachment if it exceeds 20,000 characters)

Reviewer #2: Ensure that findings will resonate with the present time.

*Thank you for your comment. We have added the following lines to highlight the relevance of our findings:*

*(Lines 86-87) ”Experiences of stigma continue to contribute towards a disproportionate burden of poor mental health among MSM today (Witzel et al., 2024).”*

*(Lines 651-655) “Further, MSM from ethnic minority backgrounds may face additional stigma negatively impacting mental health following the 2022/2024 global mpox clade IIb outbreak due to intersections between stigma around homosexuality and racism linked to both popular understandings and media depictions of groups most impacted (Roy, 2024; Witzel et al., 2024).”*
