# Supplementary material for: Barriers and facilitators to addressing mental health needs among Asian, Black and Latin American men who have sex with men (MSM) in England and Wales: A qualitative study
Source: PLOS Ment Health. 2025 Mar 27;2(3):e0000231. doi: 10.1371/journal.pmen.0000231 (PMC12798512; doi:10.1371/journal.pmen.0000231)
Supplement: Attachment — Submitted filename: Ghobrial_responses.docx [file pmen.0000231.s002.docx]

**Responses to reviewer comments**

Dear Prof Reyes,

Thank you for the reviewers’ comments for this article. We have addressed the comments point by point and include a tracked and clean copy of the manuscript.

Thank you

Dr Andrew Ghobrial

_________________________________________________________

Journal requirements:

1. Please send a completed 'Competing Interests' statement, including any COIs declared by your co-authors. If you have no competing interests to declare, please state "The authors have declared that no competing interests exist". Otherwise please declare all competing interests beginning with the statement "I have read the journal's policy and the authors of this manuscript have the following competing interests:"

- Thank you for your comment. We have added the line *“"I have read the journal's policy and the authors of this manuscript have the following competing interests:"* to our competing interests statement in the submission form. This is included with the existing Competing Interests statement in the additional information of the submission stating “TCW received speaking honoraria from Gilead Sciences between 2017 and 2023. The remaining authors have no competing interests to declare.” This is also included in the declarations section at the end of the manuscript

2. Your current Financial Disclosure states, “Prof Alison J Rodger”. Please indicate by return email the full and correct funding information for your study and confirm the order in which funding contributions should appear. Please be sure to indicate whether the funders played any role in the study design, data collection and analysis, decision to publish, or preparation of the manuscript.

- Thank you for your comment. We have reviewed the Financial Disclosure statements in the additional information of the submission form and in the Declaration section at the end of the manuscript which outlines the NIHR source of funding (reference included) as follows:
  - “This manuscript presents independent research funded by the National Institute for Health and Care Research (NIHR) under the Programme Development Grants (Reference number: NIHR203298). The views expressed in this manuscript are those of the author(s) and not necessarily those of the NIHR or the Department of Health and Social Care.”
- We have also included the following line to both statements in the submission form and declaration section of the manuscript to clarify that the funder had no role in the preparation of the paper:
  - *“The funders have had no role in the conduct of the study including its design, data collection, analysis or preparation of the manuscript.”*

3. In the online submission form, you indicated that "Due to the potentially identifiable nature of the underlying data it will not be made publicly available. The senior author (TCW) will consider individual requests for access."

- Thank you for your comment. Due to their sensitive and personally identifiable nature, underlying data will not be made freely available. Requests will be considered by the LSHTM Research Ethics Committee. Please contact [ethics@lshtm.ac.uk.This](mailto:ethics@lshtm.ac.uk.This) explanation has also been added to the declarations section of the manuscript.

Please review your reference list to ensure that it is complete and correct. If you have cited papers that have been retracted, please include the rationale for doing so in the manuscript text, or remove these references and replace them with relevant current references. Any changes to the reference list should be mentioned in the rebuttal letter that accompanies your revised manuscript. If you need to cite a retracted article, indicate the article’s retracted status in the References list and also include a citation and full reference for the retraction notice.

- Thank you for your comment. The reference list has been reformatted in accordance with APA guidance. No *references were removed. The following references were added:.*

*Khanolkar, A. R. (2024). Sexual minority health inequalities — why are we unable to do more? BMC Global and Public Health, 2(1), 34.* [*https://doi.org/10.1186/s44263-024-00066-1*](https://doi.org/10.1186/s44263-024-00066-1)

*Hawkinson, D. E., Witzel, T. C., & Gafos, M. (2024). Exploring practices to enhance benefits and reduce risks of chemsex among gay, bisexual, and other men who have sex with men: A meta-ethnography. Int J Drug Policy, 127, 104398.* [*https://doi.org/10.1016/j.drugpo.2024.104398*](https://doi.org/10.1016/j.drugpo.2024.104398)

*YouGov. (2018). LGBT in Britain: Health Report. Stonewall. Retrieved 10 Oct 2024 from* [*https://www.stonewall.org.uk/system/files/lgbt_in_britain_health.pdf*](https://www.stonewall.org.uk/system/files/lgbt_in_britain_health.pdf)

*Hudson-Sharp, N., & Metcalf, H. (2016). Inequality among lesbian, gay, bisexual, and transgender groups in the UK: a review of evidence. In: National Institute of Economic and Social Research.*

Reviewers' comments:


Reviewer #1: This is a superb paper which highlights a novel approach to understanding access to mental health services for people with intersectional identities who may be marginalised due to race and sexual orientation. The findings are well contextualised to exisitng literature, presented clearly and discussed with sufficient circumspection. There are only minor typographical/stylistic changes that I can recommend. For example, using a consistent third person narrative, but otherwise the work is highly commended.

- Thank you for your comment and for your support of our manuscript. We are very pleased to hear your feedback and appreciate the time you have spend reviewing this work. We have reviewed the paper and amended typographical errors and ensured that the narrative is consistent.

Reviewer #2: Please refer to the attached file for some notes for improvement on the specific portions of the article. You have a promising research that is beneficial to the LGBTQ+ community. Please ensure the relevance of the research outcomes to the present time since the data for the study were collected during 2020. My other notes are on the attachment. All the best ahead!

- Thank you for your comments on this submission, we are very grateful for your time and your supportive review. We have responded to each individual point in the attached file below and we have updated our manuscript with tracked changes accordingly alongside the comments above. We have added a line to the discussion to explain the relevance of the data presently as detailed in the response to the commets below.

Attached file including reviewer 2 comments:

**Title:**

-Suggest that SELPHI be spelled-out in the title for clarity

- Thank you for your comment. We have removed SELPHI from the title of the paper - the full name of the trial has been detailed in the mansucript and as the analysis is not central to HIV testing we have elected to take this out of the name of the manuscript to avoid confusion and focus the work on

**General Comments**

-Ensure general compliance with APA guidelines and standards.
 -For example, the way numbers should be written must be applied consistently in the article. In the results section of the abstract, “eleven” may be written numerically, “five” is written correctly as text.

- Thank you for your comment. We have reviewed the text and corrected discrepancies in how numbers are written in compliance with APA guidance.

-Make table compliant with APA standards.

- Thank you for your comment. We have reviewed the table (Table 1: Participant demographics) and reformatted in accordance with APA guidance.

-Check how references are written and make sure they comply with APA.

- Thank you for your comment. We have revised the reference list to comply with APA guidance.

**Abstract**

-It was mentioned that a thematic analysis was employed. What specific categories and themes were emerged from the study. I can see that specific outcomes were highlighted in the results portion of the abstract. Are these already the themes? I suggest that you mention here the specific themes that were culled from the data.

- Thank you for your comment. We have included the three meta-themes identified in the data in the Abstract by adding the following line:

*“The data were organised into three meta-themes exploring 1) Background, culture and upbringing, 2) 195 Sexuality and manifestation of mental health issues, and 3) Barriers and facilitators to accessing mental health services”*

**Introduction**

-Did you find nuisances when it comes to the experiences of Asian, Black, and Latin American people when it comes to their mental health or sexual encounters? I suggest that you present dedicated reviews for these groups and mention a common ground why you are taking them altogether in this study.

- Thank you for your comment. This has been addressed in the sentences below to acknowledge the nuances and limitations when considering these differing groups together.

*“The discrepancies also illuminate limitations in evaluating significantly heterogenous groups such as ‘LGBTQ+ people’ or ‘minority ethnic groups, ’ as sexual and gender minorities face some overlapping but also many different stressors (Parks et al., 2004). Indeed, various ethnic groups have nuanced and distinct experiences of prejudice and unique protective factors (Huang & Fang, 2019; Noyola et al., 2020; Rosario et al., 2004). Despite the complexities in understanding the nuanced intersections between race, ethnicity, gender and sexual orientation, the literature is consistent in outlining that people identifying as sexual, gender and ethnic minorities have largely unmet needs, are often unreached by public health interventions and face distinct barriers to accessing support (Bao et al., 2024; Bush et al., 2016; English et al., 2018; Jardas et al., 2023; Link & Phelan, 2006; E. J. Nicholls et al., 2022; Spengler et al., 2023).”*

- We have also added the following line to support our justification for this work, in addition to addressing this limitation in the discussion section:

*“ Acknowledging the heterogeneity between these groups, this study is intended as a starting point for further work to explore more nuanced differences between ethnic, gender and sexual minorities in the UK.”*

-What previous research outcomes were advanced related to MSM? It would be good to highlight this also in your introduction to enable readers to see where you are coming from, what specific blind- or blankspots this research is attempting to fill-in.
 -It might be helpful to include also the current state of the LGBTQ+ community in England and Wales to see the developments that such locales afforded the community through the years. This can also provide a stronger foundation for the study.

- Thank you for your comment. The following sentences have been added to outline existing research which highlights the unmet need among MSM and the LGBTQ+ community:

*“LGBTQ+ people also experience distinct differences in their mental health. Bisexual people have a higher rate of mental health concern and underrepresentation compared with gay or lesbian people (Jardas et al., 2023; Link & Phelan, 2006; E. J. Nicholls et al., 2022; Spengler et al., 2023). Sexual minority (SM) men are disproportionately affected by depression and anxiety compared with SM women, although SM women have a higher risk of drug or alcohol dependency (Armstrong et al., 2022; Hickson et al., 2017; Lelutiu-Weinberger et al., 2015; Monick et al., 2023; Stevens et al., 2020; Strong et al., 2022).”*

- We have also updated the below sentences regarding the mental health inequalities and poorer access to healthcare experienced by MSM specifically in the UK.

*“It is important to note that the majority of work exploring the mental health of ethnically minoritised LGBTQ+ people has been conducted with cisgender MSM in the United States, with a dearth of evidence from other settings. Asian, Black and Latin American MSM in the UK are vastly underrepresented in health research (Hibbert et al., 2019; McNeil et al., 2012), but existing literature demonstrates pronounced physical and mental health inequalities experienced by ethnic minority MSM in the UK. (Hickson et al., 2017; Jaspal et al., 2016; Kohli et al., 2024; Stevens et al., 2020; YouGov, 2018)”*

**Methods**

-For the Data Analysis, what measures of trustworthiness were employed to the data? -What particular data analysis framework did you utilize in analyzing the findings?

- Thank you for your comment. We have included the following line in our methods section to clarify how we ensured the trustworthiness of the data and our findings:
  - *“Throughout the analysis AG, TCW and PS met to discuss the and refine the themes as a team, ensuring trustworthiness. Input was especially sought from PS as the PPI lead for the grant. This ensured the analysis was grounded in the key issues described by the participants and reflected the needs of the community.”*
- We have included a line in the Analysis part of the methods section to outline the use of a thematic framework analysis in our process.
  - *“A thematic framework analysis was undertaken to analyse the data” (line 205)*

**Results**

-You may also want to provide a table that shows the summary of the meta-themes and the sub- themes emerged from the data

- Thank you for your comment. We have added a table (Table 2: Summary of meta-themes and themes) in summary of the thematic analysis. We have reviewed it for compliance with APA guidelines.

**Discussion**

-Are the results still updated? Do they still reflect the prevailing situations? I noticed that data collection was conducted in 2020. Ensure to update certain portions if necessary to make the results attuned to the present.

- Thank you for your comment. The data are relatively recent but the state of mental health services in the UK remains unchanged if not worse for minoritised groups, this is reflected in the use contemporaneous references in the introduction which reflect the current provision of mental health services in the UK. We have including the following sentence to outline the relevance of the paper’s findings presently:

*“ Although the data were collected in 2020, the findings still continue to resonate withreflect existing key issues these groups face in accessing mental health support. cultural norms. In addition, in the UK to worsening physical and mental health outcomes for ethnic and sexual minorities continue to worsen alongside and an ongoing deterioration in mental health service provision in the UK, enhancing the relevance of this research. “*
